# Adult child socio-economic status and older parents’ psychosocial outcomes during the COVID-19 pandemic

**DOI:** 10.21203/rs.3.rs-2719897/v1

**Published:** 2023-03-28

**Authors:** K. Renata Flores Romero, Yulin Yang, Sharon H. Green, Sirena Gutierrez, Erika Meza, Jacqueline M. Torres

**Affiliations:** UC San Francisco; UC San Francisco; UC Berkeley; UC San Francisco; UC San Francisco; UC San Francisco

**Keywords:** Socio-economic status, COVID-19 pandemic, older adults, mental health, intergenerational influences

## Abstract

**Purpose:**

Older adults’ psychosocial outcomes during the COVID-19 pandemic have been inequitable by socio-economic status (SES). However, studies have focused solely on own SES, ignoring emerging evidence of the relationship between adult child SES and late-life health. We evaluated whether adult child educational attainment – a core marker of SES – is associated with older parents’ psychosocial outcomes during the pandemic.

**Methods:**

We used data from the Survey of Health, Aging, and Retirement in Europe (SHARE; 2004–2018) and the SHARE Corona Surveys (2020 and 2021). We included 15,553 respondents > 65 years who had pre-pandemic information on adult child educational attainment, self-reported mental health, and worsened mental health compared to the pre-pandemic period. We used generalized estimating equations adjusted for respondent and family-level characteristics, including respondents’ own SES.

**Results:**

Older adults whose adult children averaged levels of educational attainment at or above (vs. below) their country-specific mean had a lower prevalence of nervousness (Prevalence Ratio [PR]: 0.95, 95% Confidence Interval [CI]: 0.91, 0.99), depression (PR: 0.96, 95% CI: 0.92, 1.00), and trouble sleeping (PR: 0.96, 95% CI: 0.92, 1.00) during the pandemic; associations with loneliness were null. Overall associations with worsened mental health as compared to the pre-pandemic period were null. Protective associations were stronger in countries experiencing “high” levels of COVID-19 intensity.

**Conclusions:**

Adult child SES may be an important driver of inequities in older adults’ mental health during the COVID-19 pandemic. Policies aimed at improving adult child SES may buffer the adverse psychosocial impacts of societal stressors.

## Introduction

Older adults have faced adverse consequences of the COVID-19 pandemic resulting from the direct threats of infection as well as disruption to social life. These and other challenges have contributed to poorer mental wellbeing among older adults.^[1–3]^ Nevertheless, recent studies have indicated that the psychosocial consequences of the COVID-19 pandemic have been inequitable across socio-economic strata, with the poorest outcomes concentrated among older adults with lower levels of education and other markers of socio-economic disadvantage.^[1,3]^ However, this research has exclusively taken an individualistic approach by focusing on the impact of own socio-economic status, ignoring growing evidence that the socio-economic status (SES) of one’s family – and most notably, one’s adult children –can influence late-life psychosocial and related health outcomes.^[4–14]^

Research from the pre-pandemic period suggests multiple overlapping mechanisms by which older adult health, including psychosocial wellbeing, might be influenced by adult child SES.^[4,5,8]^ For example, adult children with higher SES may provide critical economic and non-economic resources to older parents, including direct financial transfers or resource sharing (e.g. sharing a household).^[14]^ During the pandemic, adult child economic resources may have been used to help older adults overcome pandemic-related challenges^[15]^, like accessing testing resources or technology needed for medical appointments, and to maintain social connections with non-resident family and friends. Higher SES among adult children has also been linked in pre-pandemic research to lower levels of stress and worry for older parents (e.g. via reduced financial strain, improved relationship quality, or the exchange of social and instrumental support) as well as greater life satisfaction and quality of life;^[16–18]^ these factors may have contributed to better mental health for older parents during the pandemic.

In the present study, we bridge recent literature on socio-economic inequalities in psychosocial outcomes among older adults during the COVID-19 pandemic with pre-pandemic literature on the “upstream” influences of adult child SES on older parents’ health. We evaluated whether older European adults’ psychosocial outcomes during the COVID-19 pandemic were patterned by adult child SES independent of respondents’ own lifecourse SES. We expected that higher adult child SES would be associated with better psychosocial outcomes and lower risk of worsened mental health during the pandemic as compared to the pre-pandemic period. We secondarily evaluated associations between adult child educational attainment and older parents’ frequency of contact with children, the prevalence of help with basic needs given to and received from children, and respondents’ COVID-19-specific outcomes. These latter outcomes could serve as potential pathways of influence between adult child educational attainment and parents’ psychosocial outcomes.

Finally, we evaluated whether associations between adult child education and older adults’ psychosocial outcomes varied across both time and pandemic stage. Specifically, we expected that adult child SES could have been more impactful for parents’ outcomes during the earliest months of COVID-19, when resources to cope with the impacts of lockdown may have been particularly scarce. We also considered that there could be heterogeneity based on the intensity of COVID-19 infections, given that European countries experienced the pandemic differently at any given point in time.^[1]^

## Methods

### Data

Data for this study comes from the Survey of Health, Ageing and Retirement in Europe (SHARE)^[19]^ and the SHARE Corona Survey (SCS).^[20,21]^ SHARE has collected data on adults > 50 years of age and their spouses since 2004 with approximately biennial follow-ups through early 2020; 28 European countries and Israel have been included in SHARE. The SCS was conducted among ongoing SHARE participants via computer-assisted telephone interviews (CATI) during the early months of the pandemic (June-August 2020) and one year later (June-August 2021).

We restricted the analytic sample to respondents > 65 years in European countries (excluding Israel) who 1) had at least one living child aged 25 years and older (we excluded those with only children under 25 years of age because they may not have completed their educational trajectory), 2) reported on their child characteristics at any of the pre-pandemic SHARE waves, and 3) had completed SHARE wave 8 (conducted between October 2019 and March 2020), from which we derived our pre-pandemic control variables. and had complete data for at least one SCS wave. After applying these inclusion criteria, we were left with an analytic sample of 17,024 respondents (see eFig. 1).

### Outcome Measures

#### Psychosocial Outcomes

At each of the two SCS waves, participants answered the following questions, which each had binary response options: “In the last month, have you felt nervous, anxious, or on edge?”, “In the last month, have you been sad or depressed?”, “Have you had trouble sleeping recently?”. Respondents also reported on the frequency of feeling lonely; we grouped those who reported often or some of the time (vs. hardly ever or never).

We separately evaluated associations with self-reported worsened mental health as compared to the pre-pandemic period. During the first SCS wave, respondents were asked to rate whether each of the symptoms listed above (nervousness, depression, trouble sleeping, and loneliness) had worsened, improved, or had stayed the same as compared to the pre-pandemic period. We contrasted those who reported that their symptoms had worsened with those who reported that their symptoms had improved, stayed the same, or that they had not experienced that symptom.

#### Secondary Outcomes

Contact with children was measured at each pandemic wave with the following question: “Since the outbreak of Corona, how often did you have personal contact, that is, face to face with your own children from outside your home?”. We grouped those who responded daily, several times a week, or about once a week (vs. less often or never).

Support given to and received from children was measured with two questions at each wave: “How often did your own children help you to obtain necessities, compared to before the outbreak of Corona? Less often, about the same, or more often?”. “Compared to before the outbreak of Corona, how often did you help your own children to obtain necessities: less often, about the same, or more often?” Wording for the latter two questions shifted slightly during the second SCS wave (see Supplemental Appendix for specific question wording). We grouped those who responded less often or about the same (vs. more often).

Respondents’ COVID-19 experiences were captured with the following questions at each wave, which we evaluated as separate outcomes: “Have you or anyone close to you been tested for the Corona virus and the result was positive, meaning that the person had the Covid disease? (Yes/No)”, Have you or anyone close to you been hospitalized due to an infection from the Corona virus? (Yes/No)”, “Has anyone close to you died due to an infection from the Corona virus? (Yes/No)”.

#### Adult Child Socioeconomic Status

At the pre-pandemic SHARE waves, respondents reported the level of educational attainment for each of their children > 16 years of age. Levels of educational attainment were standardized across SHARE countries using the International Standard Classification of Education (ISCED-1997),^[22]^ with values that ranged from 0 (pre-primary education) to 6 (doctoral studies). For each respondent, we calculated a binary variable contrasting those who reported that their adult children had completed a mean level of educational attainment at or above vs. below the mean level for all respondents residing in the same country. We evaluated the consistency of our results using a continuous measure of the average level of educational attainment across adult children (range: 0–6) as well as a categorical variable based on country-specific quartiles of average adult child educational attainment.

#### Confounders and Effect Modifiers

We considered confounders to be measures that may have influenced adult-child educational attainment and older parents’ psychosocial outcomes during the COVID-19 pandemic. These included respondent’s age, gender, educational attainment, nativity, country, marital status, parents’ level of educational attainment (for mother and father), spouse’s age (if currently married/partnered), spouse’s educational attainment (if currently married/partnered or formerly married), the total number of respondent’s living children, and the percentage of female children. All these measures were captured during pre-pandemic waves of data collection. We did not control for respondents’ pre-pandemic health, given that this may have mediated the relationship between adult child educational attainment and mental health during the pandemic.

We considered variation in associations by SCS wave, which corresponded to some of the earliest months of the pandemic (Wave 1) and the post-vaccination period (Wave 2). We also considered heterogeneity by the intensity of the COVID-19 pandemic since higher COVID intensity at the time of the interview was associated with larger declines in mental health in SHARE.^[1]^ COVID-19 intensity was measured as the number of cases per 1,000 population averaged separately for each country during the three-month data collection period for the first SCS wave; data were obtained via Our World in Data, sourced from Johns Hopkins University.^[23]^ We transformed this measure into a binary indicator of cases per 1000 population at or above vs. below the median across the countries included in our analyses. We focused our analysis on heterogeneity by COVID-19 intensity on the first SCS wave (June – August 2020); given the vast differences in the landscape of vaccination and treatment by the second SCS wave (June– August 2021), the meaning of “high” COVID-19 intensity may have varied substantially by this time.

#### Analytic Strategy

We used generalized estimating equations with a Poisson distribution and a log link to analyze the associations between adult child educational attainment and older parents’ psychosocial outcomes with data pooled across both pandemic waves. While our primary models combine all respondents, we also test for differences in associations by both the gender of the adult child and respondent given mixed prior evidence of heterogeneity in studies of pre-pandemic health outcomes.^[6,10,12,14]^ We subsequently test whether these associations varied across study waves or country-level COVID-19 intensity (high vs. low) with stratified models as well as multiplicative interaction terms in pooled models. We used the same modeling strategy to estimate overall associations with secondary outcomes.

## Results

### Descriptive Statistics

Respondents’ characteristics are summarized for the first (Table 1) and second (Appendix Table 1) waves of the SCS. In the first wave, respondents were an average of 75 years of age (± 6.9 SD), primarily female (56%), born in their country of residency (93%), and married (72%). Respondents and their spouses had an average ISCED level of 2.9 (± 1.4 SD) and 2.8 (± 1.4 SD), respectively, roughly corresponding to lower secondary educational attainment. On average, respondents had two (±1.1 SD) adult children with a mean ISCED level of 3.8 (±1.0 SD), corresponding to an upper secondary level of education.

Across the two SCS waves (Wave 1 and Wave 2), 29% and 32% of respondents reported feeling nervous, 25% and 30% reported depression, 28% and 32% had sleep problems, and 28% and 30% felt lonely. In the first wave, one-fifth of respondents reported worsened nervousness compared to before the COVID-19 outbreak, 16% reported worsened depression, 8% reported worsened sleep problems, and 11% felt worsened loneliness.

In addition, across SCS Wave 1 and Wave 2, 49% and 32% of respondents reported little or no contact with their children, 67% and 37% received help from children to obtain basic necessities, and 17% and 10% reported helping their children obtain basic necessities. Finally, 6% and 36% reported a positive COVID-19 test, 3% and 12% reported COVID-19-related hospitalization for themselves or someone close to them, and 3% and 8% reported that someone close to them had died of COVID-19.

### Associations with Psychosocial Outcomes during the COVID-19 Pandemic

Respondents for whom average adult child educational attainment was at or above (vs. below) the country-specific mean had a lower risk of nervousness (Prevalence Ratio [PR]: 0.95, 95% Confidence Interval [CI]: 0.91, 0.99), depression (PR: 0.96, 95% CI: 0.92, 1.00), and sleep problems (PR: 0.96, 95% CI:0.92, 1.00) during the pandemic; the association with loneliness (PR: 0.98, 95% CI: 0.94, 1.03) was null. Results were substantively similar when using the continuous and categorical measures of adult child educational attainment (Table 2).

**Figure 1:**
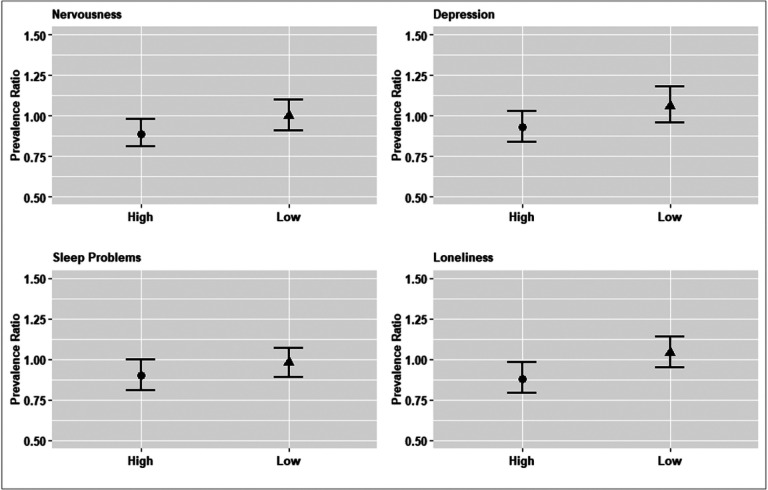
Adult Child Educational Attainment and Older Parents’ Psychosocial Outcomes, Stratified by COVID-19 Intensity, in the Survey on Health, Aging and Retirement in Europe. Notes: Nervousness (p-values: 0.017 and 0.946, respectively); Depression (p-values: 0.160 and 0.240, respectively); Sleep Problems (p-values: 0.058 and 0.581, respectively); Loneliness (p-values: 0.018 and 0.374, respectively).

There was limited evidence of heterogeneous associations by SCS wave (Appendix Table 2). However, higher adult child educational attainment was more strongly associated with a lower prevalence of nervousness (PR for interaction: 0.89, 95% CI: 0.78, 1.00, p = 0.056) and loneliness (PR for interaction: 0.87, 95% CI: 0.77, 0.99, p = 0.032) in countries experiencing higher levels of COVID-19 intensity during the first SCS wave (Appendix Table 3, Fig. 1). Associations in countries with lower COVID-19 intensity were generally null.There was limited evidence of heterogeneity by adult child gender (Appendix Table 4). While 95% confidence intervals overlapped for estimates stratified by parent gender (Appendix Table 5), the magnitude of associations was larger for fathers (vs. mothers; associations for mothers were null).

### Associations with Worsened Psychosocial Outcomes Compared to the Pre-Pandemic Period

**Figure 2:**
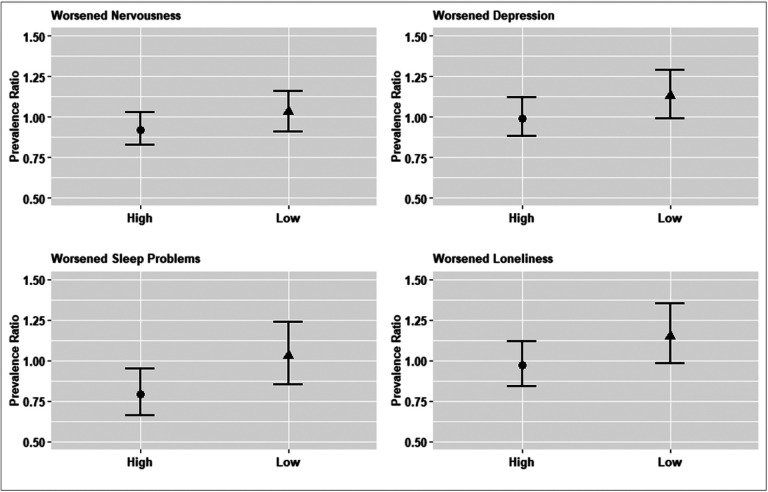
Adult Child Educational Attainment and Older Parents’ Psychosocial Outcomes Compared to the Pre-Pandemic Period, Stratified by COVID-19 Intensity, in the Survey on Health, Aging and Retirement in Europe. Notes: Nervousness (p-values: 0.145 and 0.629, respectively); Depression (p-values: 0.890 and 0.078, respectively); Sleep Problems (p-values: 0.013 and 0.759, respectively); Loneliness (p-values: 0.714 and 0.095, respectively).

We found no evidence of overall associations between adult child educational attainment and respondents’ self-assessments of worsened mental health as compared to the pre-pandemic period (Appendix Table 6). There was once again evidence of heterogeneity by country-level COVID intensity: higher adult child educational attainment was associated with a lower prevalence of worsened nervousness (PR for interaction: 0.85, 95% CI: 0.74, 0.99, p = 0.035) in countries experiencing higher COVID-19 intensity during the first SCS wave (Appendix Table 7, Fig. 2). Associations in countries with low COVID-19 intensity were null (for worsened nervousness or sleep problems) or in opposite direction from hypothesized (for worsened depression and loneliness).

There is no evidence of heterogeneity by adult child gender (Appendix Table 8). While 95% confidence intervals overlapped throughout, associations between higher adult child educational attainment and worsened depression and loneliness were of larger magnitude for mothers (vs. fathers) and in the opposite direction from hypothesized (Appendix Table 9).

### Associations with Potential Family, and COVID-related Mechanisms

Average adult child educational attainment at or above (vs. below) the country-level mean was associated with a higher risk of having little or no in-person contact with children (PR: 1.07, 95% CI: 1.03,1.11) (Appendix Table 10). Associations with either receiving help from or giving help to children to obtain necessities were generally null.

Higher average adult child educational attainment was associated with a higher risk of testing positive for COVID-19 or having someone close that tested positive (PR: 1.09, 95% CI: 1.04, 1.14), but not with having been hospitalized, knowing someone close that had been hospitalized with COVID-19, or having someone close that died of COVID-19 (Appendix Table 11). All results were substantively similar when using binary, continuous, and categorical measures of adult child educational attainment (Appendix Table 12 and 13).

## Discussion

In a population-based study of older European adults, we found that higher adult child educational attainment – a core marker of socio-economic status – was associated with a lower risk of poor mental health outcomes during the COVID-19 pandemic, including nervousness, depression, and trouble sleeping. This study extends growing evidence based on pre-pandemic data suggesting the importance of adult child socio-economic status for older parents’ health,^[6–9,17]^ but is the first to focus on parents’ outcomes during the COVID-19 pandemic.

Contrary to our hypotheses, associations between adult child educational attainment and parents’ selfreports of worsened mental health as compared to the pre-pandemic were null overall. Nevertheless, we found important heterogeneity by country-level COVID-19 intensity. In models focused on the first SCS wave, we found that associations between high adult child educational attainment and respondents’ risk of poor mental health as well as worsened nervousness compared to the pre-pandemic period were of stronger magnitude in countries experiencing a higher number of COVID cases during the survey period. These findings could suggest that adult child socio-economic resources may be most important for older parents’ wellbeing during periods of societal and health-related duress. Fundamental cause theory^[24]^ suggests that higher socio-economic status can provide individuals with resources that can be used flexibly to prevent the direst consequences of challenging life circumstances. We did not find evidence of heterogeneity by SCS wave, which may suggest that the specific pandemic-related country-level circumstances may have been a more important modifier than a broad time frame.

We additionally found that higher adult child educational attainment was associated with less in-person contact with children across the pandemic waves and a higher risk of reporting COVID-19 infection among oneself and/or someone close. These findings have little precedent in the pre-pandemic literature. One prior study found evidence of an association between higher adult daughter educational attainment and more frequent child contact for older parents in South Korea.^[14]^ Our contrary findings may have been driven by pandemic-specific circumstances. For example, it may have been the case that higher SES adult children may have been less likely to co-reside with older parents, which would have limited opportunities for contact during a time of pandemic-related physical distancing. In addition, higher SES children may have been more likely to have received information about the risks of in-person contact for COVID-19 transmission among older parents. The association between higher adult child educational attainment and higher risk of experiencing a COVID-19 infection could be an artifact of greater testing resources among higher SES families or higher risk in some occupations that require higher levels of education (e.g. medical professionals) during the early pandemic period. Future research may further pinpoint the mechanisms underlying these results.

Finally, we found little systematic evidence of heterogeneity in associations by adult child gender. There was evidence of differences in the magnitude of association by respondent gender, although patterns varied by outcomes and confidence intervals overlapped. Prior studies have been extremely mixed in this regard. For example, some studies have found greater health returns to adult child education for mothers^[6,11]^ while others have reported associations for fathers only,^[10]^ while others have reported no evidence of heterogeneity.^[16,25]^ Limited evidence of heterogeneity may point to the consistent importance of adult child educational attainment

### Limitations

We acknowledge important study limitations, including residual confounding due to respondents’ longstanding pre-pandemic economic and health conditions that may have influenced both adult child educational attainment and pandemic outcomes. In addition, while educational attainment may be an important driver of contemporaneous

SES, more proximate dimensions of adult child SES – including current income and wealth^26^ – were not available. Finally, we could not evaluate heterogeneity by adult child co-residence or consider whether patterns might be explained by co-residence dynamics,^[27]^ since the co-residence status of specific adult children was not evaluated during the pandemic waves.

## Conclusion

In this population-based study of older European adults, adult child educational attainment measured before the pandemic was associated with older parents’ mental health during the pandemic. Associations with mental health and worsened mental health (vs. the pre-pandemic period) were stronger within countries experiencing higher COVID-19 intensity as measured by case counts. Adult child educational attainment was also associated with other parental outcomes during the pandemic, albeit in unexpected ways: a lower risk of in-person contacts with adult children and a higher risk of experience with COVID-19 infection. This is the first study, to our knowledge, to empirically consider the role of adult child socio-economic status in shaping older parents’ outcomes during the COVID-19 pandemic. Our findings suggest that policies that increase educational attainment may have “upward” spillover effects on the psychosocial wellbeing of older parents, particularly during periods of societal duress.

## Figures and Tables

**Table 1.** Descriptive Characteristics, Older European Adults 65+ years in the Survey on Health, Aging and Retirement in Europe (SHARE) and SHARE Corona Survey, June-August 2020 (N=14,782)

	Mean (SD)	N (%)
**Pre-COVID Socio-Demographic Characteristics**		
Age, mean (SD)	74.8(6.9)	
Female, n (%)		8263(55.9)
Native-born, n (%)		13711(92.8)
Marital status, n (%)		
Married/partnered		10587(71.6)
Widowed		2,806(19.0)
Divorced/separated		1168(7.9)
Single		221(1.5)
Average level of educational attainment (range: 0–6), mean (SD)	2.9(1.4)	
**Pre-COVID Respondent Characteristics**		
Average level of educational attainment for mother (range: 0–6), mean (SD)	1.4(1.2)	
Average level of educational attainment for father (range: 0–6), mean (SD)	1.7(1.4)	
**Pre-COVID Spouse Characteristics**		
Age of current spouse (if married/partnered), mean (SD)	73.3(7.2)	
Average level of educational attainment for current or former spouse (range: 0–6), mean (SD)	2.8(1.4)	
**Pre-COVID Child Characteristics**		
Total number of children, mean (SD)	2.4(1.1)	
Female children, mean (SD)	49.0(36.1)	
Average level of educational attainment among all children (range: 0–6), mean (SD)^a^	3.8(1.0)	
**Outcomes and Experiences, June-August, 2020**		
**Covid Intensity**		
Covid cases per thousand population, mean (SD)^b^	232(176.6)	
**Mental Health, n (%)**		
Nervous		4224(28.6)
Depressed		3740(25.3)
Trouble sleeping		4170(28.2)
Often lonely		4083(27.6)
Worsened nervousness		2986(20.2)
Worsened depression		2382(16.1)
Worsened trouble sleeping		1137(7.7)
Worsened often lonely		1671(11.3)
**Family Relationships and Contact, n (%)**		
Little or no contact with children		7184(48.6)
Received help from child to obtain necessities since the outbreak		3332(67.4)
Helped child to obtain necessities since the outbreak		173(17.2)
**Overall Covid Outcomes, n (%)**		
Anyone tested positive for COVID-19		892(6.0)
Anyone hospitalized due to COVID-19		451(3.1)
Anyone died due to COVID-19		371(2.5)

Source: Survey on Health, Aging and Retirement in Europe and SHARE Corona Survey.

Notes:

a. Educational levels based on the International Standard Classification of Education (ISCED, 1997), with values that ranged from 0 (pre-primary education) to 6 (doctoral studies);

b. Data come from Our World in Data, sourced from Johns Hopkins University.

**Table 2.** Adult Child Educational Attainment and Older Parents’ Psychosocial Outcomes During the COVID-19 Pandemic

	Nervousness		Depression		Trouble Sleeping		Loneliness	
	PR	95% CI	PR	95% CI	PR	95% CI	PR	95% CI
High Adult Child Educational Attainment	0.95**	0.91 – 0.99	0.96*	0.92 – 1.00	0.96*	0.92 – 1.00	0.98	0.94 – 1.03
Continuous	0.96***	0.94 – 0.98	0.97***	0.95 – 0.99	0.96***	0.94 – 0.98	0.99	0.97 – 1.01
Quartiles								
1 (Reference category)								
2	0.95*	0.90 – 1.00	0.97	0.91 – 1.03	1.00	0.94 – 1.05	0.98	0.92 – 1.03
3	0.96*	0.91 – 1.00	0.96	0.91 – 1.01	0.97	0.92 – 1.02	0.98	0.93 – 1.03
4	0.91**	0.84 – 0.99	0.92*	0.85 – 1.01	0.88***	0.81 – 0.96	0.93*	0.86 – 1.01

Source: Survey on Health, Aging and Retirement in Europe and SHARE Corona Survey. Notes: Exposure is a binary indicator of average adult child educational attainment at/above vs. below the country-specific mean. Controls include age, gender, educational attainment, nativity, country, marital status, parental education (mother and father), age of current spouse (if married/partnered) and educational attainment of current or former spouse, number of children and percentage of female children.

*** *p* < 0.001,

** *p* < 0.01,

* *p* < 0.05
